# Hepaticocholecystoenterostomy as an alternative to hepaticojejunostomy for biliary bypass

**DOI:** 10.1308/003588412X13171221592294

**Published:** 2012-10

**Authors:** J Gani, K Lewis

**Affiliations:** ^1^Lake Macquarie Specialist Medical Centre,Australia; ^2^John Hunter Hospital,Australia

**Keywords:** Pancreatic Cancer, Bile duct, Biliopancreatic diversion, General surgery

## Abstract

**INTRODUCTION:**

Hepaticojejunostomy is the standard biliary bypass technique for periampullary cancer when trial dissection reveals unresectable disease or endoscopic stent placement is not possible. This anastomosis can be technically demanding and potentially difficult. The simpler technique of hepaticocholecystoenterostomy (HCE) has only previously been reported in very limited numbers and without outcome data.

**METHODS:**

All patients undergoing HCE for the management of periampullary cancer were identified from a prospectively maintained computerised database of a single surgeon and were reviewed retrospectively. The HCE technique achieves a biliary bypass by two anastomoses, using the gallbladder as a conduit. It involves an anastomosis of the infundibulum of the gallbladder to the common hepatic duct followed by a second anastomosis of the gallbladder fundus to the proximal small bowel.

**RESULTS:**

From 1996 to 2010, 30 patients with pancreatic adenocarcinoma required a biliary bypass after a failed trial of Whipple procedure (80%) or failed endoscopic stenting (20%). There were 19 men and 11 women with a mean age of 64.5 years. The mean operative time for HCE alone was 92 minutes. The mean length of hospital stay was nine days. There was a single grade 2 complication (readmission with gastric emptying delay) and a single grade 3 complication (bile leak requiring reoperation). Thirty-day mortality was zero and the mean survival was 12 months (with one patient still alive at the time of writing). There were no readmissions with recurrent biliary obstruction or cholangitis. One patient had developed an incisional hernia by the 24 month follow-up appointment.

**CONCLUSIONS:**

HCE in periampullary cancer is safe and effective in selected patients. It involves two simple anastomoses with good access rather than one more demanding anastomosis. Morbidity, patency and overall survival are comparable with contemporary published series of hepaticojejunostomy.

Sixty to eighty per cent of patients with periampullary cancer will have unresectable disease due to local invasion or metastases.^1^ Currently, most patients are palliated endoscopically but surgical bypass can provide good palliation in cases of failed stenting or when unresectable disease is discovered at the time of surgery (usually after a trial Whipple dissection).^2^

Hepaticojejunostomy (usually Roux-en-Y) is the traditional surgical biliary bypass technique. Complication rates and outcome data have been well reported.^3^ This anastomosis can be technically demanding and potentially difficult, particularly with bulky periampullary tumours, short fatty jejunal mesentery, and intra-abdominal adiposity resulting in poor visibility and difficult access. This is particularly evident in patients with an elevated body mass index.

Other techniques have been reported such as simple cholecystoenterostomy. This is inappropriate if the cystic duct insertion is low (and therefore close to or indeed into the tumour) and can fail because the cystic duct calibre is narrow and can become occluded with debris after the anastomosis has been performed. These shortcomings have led to the technique being largely abandoned in clinical practice.

We report an alternative method of surgical biliary bypass using the gallbladder as a conduit. Initially described by Weintraub *et al* in 1980,^4^ in the first reports this technique was used as a secondary procedure when cystic duct obstruction produced recurrent jaundice. Later reports described it as a primary technique when the cystic duct was found to be obstructed at the time of biliary bypass.^5^ Eventually, some surgeons advocated it as a primary technique but only 11 cases have been reported and only 6 with outcome data (Table 1).^6,7^

**Table 1:** Published literature on hepaticocholecystoenterostomy (HCE)

Authors	Number of HCE patients		Cystic duct obstructed	Follow-up duration
	1st operation	2nd operation
Weintraub *et al,* 1980^4^	0	1	1	Not stated
Garnjobst, 1982^5^	3	2	5	4–22 months
Browne, 1986^8^	Intrahepatic			
Koga and Nakayama, 1987^6^	5	0	0	4–7 months
Allan and Jackson, 1989^7^	5	1	1	Not stated
Safioleas, 2005^9^	1	0	0	8 months

Browne was first to use the term hepaticocholecystojejunostomy^8^ for a more complex operation for hilar obstruction but we have used the term hepaticocholecystoenterostomy (HCE) as it describes accurately the use of the in situ gallbladder as a conduit between the common hepatic duct and the small bowel to effect a biliary bypass. We report the results of our experience with HCE and compare these with a contemporary published study^3^ of patients undergoing hepaticojejunostomy (Table 2).

**Table 2:** Hepaticocholecystoenterostomy compared with a published series of hepaticojejunostomy

	Our study *n*=30	Sohn *et al* (1999)^3^ *n*=256
Mean age	65.5 years	64.0 years
Sex	19 males (63%)	146 males (57%)
Peri-operative mortality	0%	3.1%
30-day mortality	0%	3.1%
Mean post-operative length of stay	8.6 days	10.1 days
Mean operative time	147 minutes	234 minutes
Reoperation	3.4%	1%
Leaks	3.4%	5%
Post-operative cholangitis	0%	2%
Mean follow-up duration	10.2 months	8.7 months
Mean survival	12 months	6.5 months

## Technique

Patients with obviously incurable disease are treated usually by endoscopic techniques so most patients having open biliary bypass have undergone a trial dissection for a Whipple procedure. The gallbladder is not mobilised during this phase. If the tumour is unresectable, the gallbladder is assessed for its viability as a conduit. Essential for this purpose are good vascularity with preservation of the cystic artery, pliability without excessive contraction or fibrosis (gallbladder stones do not prevent its use in the bypass per se) and adequate mobility so that a tension free anastomosis can be undertaken to the common hepatic duct.

**Figure 1:**
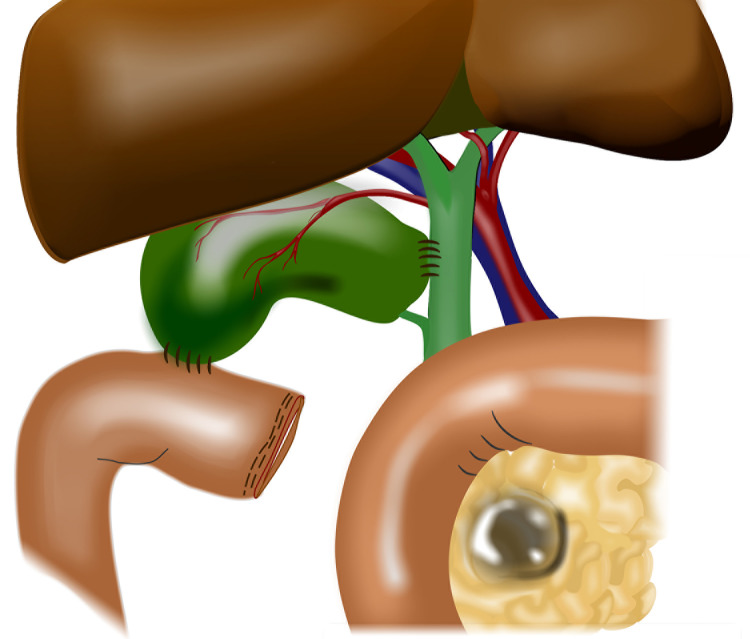
Completed hepaticocholecystoenterostomy

If these criteria are met, the gallbladder is anastomosed to the common hepatic duct usually just above the cystic duct insertion with a single layer of interrupted 4/0 polydioxanone (PDS®; Ethicon, Somerville, NJ, US). The fundus of the gallbladder is then anastomosed to either a Roux-en-Y loop or a simple jejunal loop in two layers using 4/0 PDS®. Both anastomoses are between 1.5cm and 2cm in diameter (Fig 1).

## Methods

A prospective operative database identified all patients undergoing a biliary bypass by a single surgeon working in a tertiary hospital in Newcastle, Australia, between 1996 and 2010. Over this 14-year period, 30 of the 129 patients identified underwent HCE. The medical records of these patients were reviewed retrospectively to assess the short-term morbidity and the long-term outcome (particularly anastomotic patency) of HCE. The results of our experience with HCE were then compared with a contemporary published study of patients undergoing a hepaticojejunostomy from another institution.^3^

## Results

All 30 patients in this series had a pancreatic adenocarcinoma. Twenty-four patients required a biliary bypass after a failed trial of a Whipple procedure or findings of unexpected metastatic disease and six due to failed endoscopic stenting. There were 19 men and 11 women with a mean age of 65 years. Eight of the thirty patients underwent a primary gastroenterostomy at the time of HCE, and two presented at a later date with gastric outlet obstruction and required this subsequently. Roux-en-Y cholecystojejunostomy was performed in 24 patients and 6 underwent a simple loop cholecystoenterostomy. Operative times ranged from 65 minutes to 270 minutes. The mean operating time was 149 minutes but included a trial Whipple dissection in 24 of the 30 cases. The mean operative time for HCE alone was 92 minutes.

The mean length of hospital stay was 9 days (range: 5–17 days). There was a single grade 2 complication (readmission with gastric emptying delay 3 days after initial discharge) and a single grade 3 complication (bile leak requiring reoperation). This occurred at the inferior apex of the hepaticocholecystostomy and was due to excessive tension at this point. At reoperation on day 5, a T-tube was inserted into the leak point to create a controlled biliary fistula. The patient subsequently made an uncomplicated recovery but required a prolonged hospital stay (17 days).

Thirty-day mortality was zero. The mean follow-up duration was 10.2 months (range: 1–29 months) with a mean survival of 12 months. There were no readmissions with recurrent biliary obstruction or cholangitis. One patient had developed an incisional hernia identified by the two-year follow-up appointment (Table 3).

**Table 3:** Operative complications

Age at time of operation	Sex	Clavien–Dindo classification^10^
59 years	Male	Grade 2 delayed gastric emptying
61 years	Male	Grade 3 bile leak
68 years	Female	Incisional hernia at 2-year follow-up

## Discussion

Given that only 20% of pancreatic cancer is resectable at the time of presentation, surgical bypass remains important in palliating patients who have been found at a trial dissection for a Whipple procedure to have unresectable disease. It can also be used in selected patients when endoscopic biliary stenting fails as an alternative to percutaneous transhepatic stent placement.

Compared with a standard Roux-en-Y hepaticojejunostomy, which is often difficult in the presence of a bulky periampullary tumour, HCE provides both good access and visibility (to facilitate the anastomoses). It does, however, require two anastomoses and while this may be perceived as a potential disadvantage, both anastomoses are usually easily visible and readily accessible; these anastomoses are therefore relatively easy to perform. The importance of avoiding tension in the anastomosis is highlighted by the single bile leak we report. Improving the utility of this procedure by mobilising the hepatic aspect of the gallbladder may help avoid this problem but does risk devascularising the conduit.

The presence of stones in the gallbladder per se does not prevent it being used as a conduit for HCE. Previously, there have been reports of stone formation in the gallbladder when used as a conduit but in our experience it was not problematic in this group of palliative patients.^11^

## Conclusions

Our data show that HCE is an effective and durable bypass. (There were no cases of recurrent biliary obstruction.) It is a safe treatment for biliary obstruction with survival and complication rates comparable with hepaticojejunostomy. Hepaticojejunostomy has been the gold standard for biliary bypass and will continue to provide excellent palliation for patients with locally advanced and metastatic periampullary cancer. Nevertheless, we commend the HCE technique to all abdominal surgeons as a simple and easy technique that should appeal to both specialist and non-specialist hepatobiliary surgeons alike.

## References

[1] Verslype C, Van Cutsem E, Dicato M*et al* The management of pancreatic cancer. Current expert opinion and recommendations derived from the 8th World Congress on Gastrointestinal Cancer, Barcelona, 2006. Ann Oncol 2007; 18 Supplement 7: vii1–vii10 17600091 10.1093/annonc/mdm210

[2] House MG, Choti MA. Palliative therapy for pancreatic/biliary cancer. Surg Clin N Am 2005; 85: 359–371 15833477 10.1016/j.suc.2005.01.022

[3] Sohn TA, Lillemoe KD, Cameron JL*et al* Surgical palliation of unresectable periampullary adenocarcinoma in the 1990s. J Am Coll Surg 1999; 188: 658–666 10359359 10.1016/s1072-7515(99)00049-6

[4] Weintraub MD, Grünspan M, Singer D. Hepaticocholecystostomy and cholecystojejunostomy for bile drainage. A palliative procedure. Am J Surg 1980; 139: 441–442 6153869 10.1016/0002-9610(80)90311-6

[5] Garnjobst W. Hepatocholecystostomy complemental to palliative cholecystoenterostomy. Arch Surg 1982; 117: 1,246–1,247 10.1001/archsurg.1982.01380330100026 6180700

[6] Koga A, Nakayama F. One stage hepaticocholecystojejunostomy as an easy and long term effective bilioenteric bypass for unresectable carcinoma of the pancreas. Surg Gynecol Obstet 1987; 165: 177–179 2440122

[7] Allan SM, Jackson DB. The gallbladder as a bilioenteric conduit. Ann R Coll Surg Engl 1990; 72: 22 PMC2499076 2301898

[8] Browne MK. Hepaticocholecystojejunostomy as a simple technique for decompression of the biliary tract. Surg Gynecol Obstet 1986; 163: 174–176 2426813

[9] Safioleas MC. A new technique for the palliative treatment of high bile duct cancer. Hepatogastroenterology 2005; 52: 378–380 15816440

[10] Dindo A, Demartines N, Clavien PA. Classification of surgical complications: a new proposal with evaluation in a cohort of 6336 patients and results of a survey. Ann Surg 2004; 240: 205–213 15273542 10.1097/01.sla.0000133083.54934.ae PMC1360123

[11] Wojcicki M, Milkiewicz P, Silva M. Biliary tract complications after liver transplantation: a review. Dig Surg 2008; 25: 245–257 18628624 10.1159/000144653

